# The Effect of Yoga Meditation Practice on Young Adults’ Inhibitory Control: An fNIRS Study

**DOI:** 10.3389/fnhum.2021.725233

**Published:** 2021-09-13

**Authors:** Dongdong Jiang, Zongyu Liu, Guoxiao Sun

**Affiliations:** School of Physical Education, Shandong University, Jinan, China

**Keywords:** yoga meditation, young adults, inhibitory control, fNIRS, Flanker tasks

## Abstract

**Objectives:** The present study aimed to test the effect of yoga meditation (YoMed) practice on inhibitory control of young adults.

**Methods:** A total of 50 participants (23 male, 21–28 years old) from a university in Jinan, Shandong Province were enrolled in this study. Participants were randomly assigned to a YoMed group or a Control group. Participants’ basic information, physical activity, and inhibitory control were measured. A multi-channel continuous-wave near-infrared spectrometer was used to monitor the brain’s hemodynamic responses.

**Results:** After the intervention, we found significant differences in Flanker tasks between the YoMed group and Control group. The accuracy in the YoMed group was higher than those in the Control group (*p* < 0.05). Analysis of fNIRS data showed that oxyhemoglobin (oxy-Hb) levels in the prefrontal cortex (PFC) increased in the YoMed group during the Flanker tasks after the YoMed intervention.

**Conclusion:** YoMed has a temporarily promoting effect on the brain activation of young adults. It is an effective and appropriate exercise to improve the inhibitory control of young adults.

## Introduction

Cognitive function is the mental activity or process of using thought, experience, and feeling to obtain knowledge and understanding, including attention, working memory, language, spatial orientation, and execution (Van de Geer and Jaspars, 1966; Larsen et al., 2020; Liu et al., 2020). It plays an important role in the performance of young adults who are in the critical period of growth and development (Sharma et al., 2020). Chang et al. (2020) considered that cognitive function is a strong predictor of improvement in physical function, which is critical to optimal daily functioning and can affect physical and mental health, learning, and attention levels for other activities (El-Malky et al., 2015). Blair and Raver (2014) have proved that as a part of cognitive function, the executive function can be a strong predictor of academic success. Executive function includes working memory, inhibition, and cognitive flexibility (Miyake et al., 2000), which are essential elements of higher-level processes such as reasoning and problem solving (Amunts et al., 2021). Inhibitory control is significantly associated with mental health and is considered a core component of executive function, which is important for all cognitive processing (Smith and Jonides, 1999). Research has shown that executive function is associated with young adults’ mental health and academic achievement, and inhibitory control (a critical component of executive function) is associated with their healthy habits (Diamond, 2013; Ludyga et al., 2019; Xie et al., 2020). Therefore, it is meaningful work to identify the factors that enhance inhibitory control (Frith et al., 2017; Loprinzi and Lovorn, 2019; Zagaria et al., 2021).

Meditation is defined as a form of mental training designed to improve an individual’s core mental abilities, such as self-regulation of attention and emotion (Tang et al., 2015). It has been shown to improve various parts of executive function (Tang et al., 2015; Luu and Hall, 2017). A study conducted by Sankar et al. (2020) found that cognitive abilities were enhanced in people who practiced meditation. Meditation is considered to be a feasible, acceptable, and cost-effective intervention for the benefit of cognitive and psychological symptoms in breast cancer survivors (Henneghan et al., 2020). The study conducted by Spadaro and Hunker (2016) used a remote meditation intervention that was easy to learn, required little instruction, and easily integrated into the participants’ busy schedule (only 12 min per day), showing some effectiveness in reducing stress and anxiety and improving the cognitive performance of nursing students. Research by Campos et al. (2019) also confirmed that certain traits (such as empathy and emotion recognition) are better in meditators than in non-meditators. A neurological study of adults shows that regular practice of meditation may have a protective effect on nerves and reduce the decline of cognitive ability caused by normal aging (Pagnoni and Cekic, 2007). The research on mindfulness meditation practice of high school students suggested that meditation has intrinsic perceived benefits, which can help youth change their thoughts, behaviors, and emotions (Wisner and Starzec, 2016). Previous studies of adults using meditation have shown that participating in short-term meditation programs can improve executive function and cognitive efficiency in different areas of the brain on attentional tasks (Bueno et al., 2015; Taren et al., 2017).

Meditation activities can be divided into focused-attention (FA) and open-monitoring (OM) meditation which have different effects on brain activity (Lutz et al., 2008). FA meditation requires meditators to focus their attention on a specific object or goal. When participants’ attention leaves the center of the object, they need to return to their previous state of concentration (Lutz et al., 2008). OM meditation does not require the focus of attention on a specific object. It emphasizes maintaining the monitoring of the things currently experienced (specific feelings, thoughts, emotions, etc.) in a non-judgmental attitude, which is a state of awareness (Lutz et al., 2008). Basic meditation techniques include transcendental meditation, mindfulness-based stress reduction (MBSR), and so-called yoga meditation (YoMed) Kirtan Kriya (KK) – all of which can have a positive impact on cognitive function (Pomykala et al., 2012). As a subset of meditation practice (Ospina et al., 2007), YoMed belongs to FA meditation (Lutz et al., 2008). The YoMed used in this study comes from Astanga yoga (one of the most famous YoMed systems), which was performed in a seated posture during meditation (Taimini, 1961; Amin et al., 2014). Studies have shown that YoMed can improve cognitive function. Lemay et al. (2019) have shown that practicing YoMed once a week can reduce stress and anxiety in students. Saoji et al. (2017) found that YoMed had a positive effect on college medical students’ performance on cognitive tasks. An fMRI study of YoMed found that YoMed is associated with sustained attention, memory, semantic recognition, creativity, and enhanced dissociation (Nilkamal Singh, 2014). A brief daily YoMed practice can improve mental and cognitive function and reduce depressive symptoms (Lavretsky et al., 2013). The yoga meditators had more brain activation in the PFC during the Stroop tasks than those who did not receive the YoMed intervention in a matched control group (Froeliger et al., 2012b). Dodich et al. (2019) found that YoMed sessions can have a significant impact on the brain structure of attention, one of the cognitive functions. The practice of YoMed may lead to the promotion of lasting change in the field of cognitive control, resulting in overall function enhancement (Froeliger et al., 2012a).

The functional near-infrared spectroscopy (fNIRS), which assesses brain activity, is a technology that detects changes in the concentration of oxygenated and deoxygenated hemoglobin molecules in the blood (Sonkaya, 2018). It indirectly assesses neuronal activity by measuring changes in oxy-Hb and deoxyhemoglobin (deoxy-Hb) in tissue using near-infrared light, and can further predict neural activity in the cerebral cortex (Kim et al., 2017). Jobsis (1977) observed the hemodynamic changes in animal brain for the first time using fNIRS. As a kind of brain neuroimaging technology that dynamically monitors the activity of nerve cells, fNIRS has the advantages of portability, non-invasive and continuous monitoring (Gervain et al., 2011). It takes blood volume and blood oxygen in brain tissue as the information carrier, and monitor brain activity by measuring changes in oxygen-containing hemoglobin in the cognitive process of the brain, so as to study the neural mechanism of cognitive activities (Hoshi, 2003). It is worth noting that most of the studies about the effects of YoMed on the behavioral performance of individual executive function exist (Sharma et al., 2014; Luu and Hall, 2017), while the cerebral neural mechanism of how YoMed intervention improves individual cognitive function still need to be investigated. As a non-invasive technique, fNIRS is suitable to measure the cerebral blood oxygen mechanism, however, few studies have used it to measure the PFC of young adults to find the cognitive benefits of YoMed practice. Therefore, it is necessary to use fNIRS to measure cerebral blood oxygen during young adults’ cognitive tasks, and use the changes of blood oxygen to evaluate the effects of YoMed practice on the activities of neurons in the PFC.

Based on this, the present study used fNIRS to monitor the PFC oxygenation of participants (young adults) during cognitive tasks. The purpose of this study is to explore the cerebral blood oxygen mechanism of YoMed to improve executive function and to provide a practical basis for improving executive function in young people. Based on the existing research results, this study proposes the following hypotheses: (1) YoMed practice was related to the positive behavior of executive functional tasks, which was embodied in accuracy of the flanker task; (2) YoMed practice was associated with an increase in oxy-Hb concentration in the brain during’ the inhibition task.

## Materials and Methods

### Participants

A total of 50 participants (23 male, 21–28 years old with a mean age of 23.82, and SD = 1.30) from a university in Jinan, Shandong, were included in this study. The participants were randomly divided into two groups. The YoMed group consisted of 25 participants (11 male), and the Control group consisted of 25 participants (12 male). Participants were excluded if they had YoMed practice experience or brain injury. The questionnaire obtained the basic information of participants (including height, weight, and other demographic indicators). All procedures were approved by the Ethics committee. Informed consent was obtained from all participants before study enrollment.

### Materials

#### Physical Activity Rating Scale (PARS-3)

The physical activity was assessed using the Physical Activity Rating Scale-3 (PARS-3) revised by Liang and Liu (1994), which is used to measure intensity, activity time, and frequency of physical activity. A 5-point Likert scale was used for quantification, with each item scored on a scale of 1–5. Physical activity score = activity intensity score×(activity time score−1)×activity frequency score, score interval was from 0 to 100 points.

#### Measurements of Cognitive Executive

The participants’ performance on executive functions was measured by a computerized Flanker task. The task was programed using E-Prime (Psychology Software Tools, Version 3.0) in an event-related design.

**FIGURE 1:**
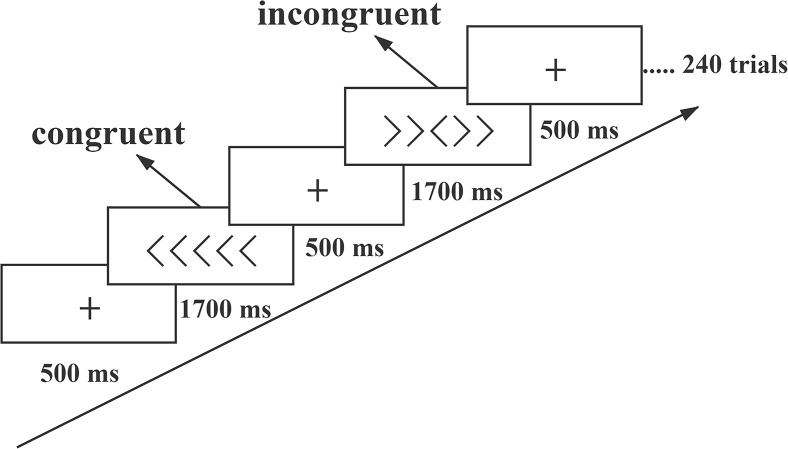
The Flanker task paradigm. The full Flanker task consists of 4 blocks of 240 trials (120 incongruent, 120 congruent). The stimuli are randomly presented in the center of the computer screen. Each block has a 10 s rest period.

The formal task consisted of 240 trials (120 incongruent, 120 congruent), which were presented in four blocks with a 10 s rest between blocks. Here, participants were asked to classify a central target symbol as “<” or “>” as quickly as possible while ignoring a peripheral distractor symbol that could be either congruent (e.g., <<<<< if the target was “<”) or incongruent (e.g., >><>> if the target was “<”) with the target symbol. Classifications were made with typed keyboard responses of “F” and “J” for “<” and “>,” respectively (Figure 1). All stimuli appeared randomly, with the next stimulus appearing after a keystroke response. The software recorded reaction times (RTs) and accuracy. Before the formal test, all participants received the same 12-trial practice as familiarization.

### Functional Near-Infrared Spectroscopy (fNIRS)

**FIGURE 2:**
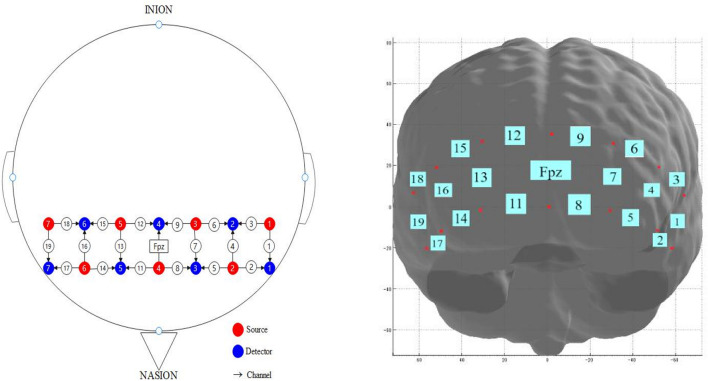
Emitter and probe configuration for functional near infrared spectroscopy (fNIRS). The fNIRS system was attached to the prefrontal area. The center of the probe matrix was placed on Fpz (International 10–20 system).

A multi-channel continuous-wave near-infrared spectrometer (NirSmart, Danyang Huichuang Medical Equipment Co., Ltd.) was used to monitor the brain’s oxygen content. The sampling rate was 10 Hz. Changes in blood oxygen concentration were recorded using two wavelengths of near-infrared light (760 and 850 nm). In our system, seven signal sources and seven detectors (probe spacing 3 cm) were placed at alternate points on a 2 × 7 grid, enabling us to detect signals from 19 channels covering the FPC (Figure 2). The center of the probe matrix was placed on Fpz (International 10–20 system). We checked the cap position before acquisition to ensure accuracy and used another cap (without a probe) to cover the collection head cap and fixed it with Velcro to ensure the signal would not be affected by external light.

### Procedures

**FIGURE 3:**
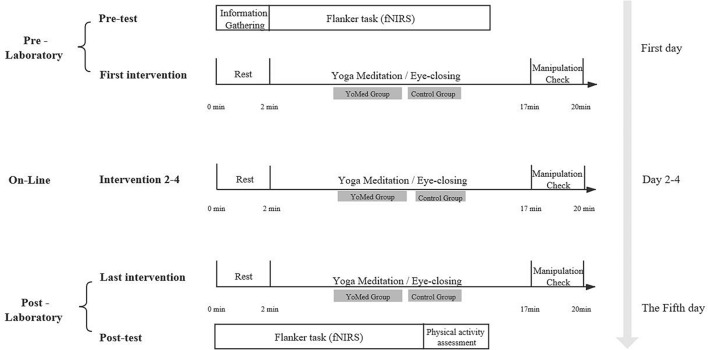
Chart of the experimental protocol.

The experimental protocols are shown in Figure 3. Participants visited the laboratory on two separate occasions, completed the Pre-test (1st day) and the Post-test (5th day). The intervention was conducted over five consecutive days (from Friday to next Tuesday) and the time (i.e., 9:00–9:15 in the morning) of each intervention was the same. And the whole experiment lasted 5 weeks. During the 5-day intervention period, the participants in the YoMed group practiced YoMed for 15 min daily by sitting in the chair, remaining silent, and listening to “YoMed guidelines” on the basis of maintaining the previous living habits, with no other activities arranged. Based on the same living habits, the participants in the Control group were given a 15-min eye-closing rest every day, with no other activities arranged. Participants were asked to wear earphones during the intervention.

Before the YoMed practice and the eye-closing rest, we use the “preparation instructions” to inform participants to be ready to enter the meditation or rest state. The “preparation instructions” used before intervention for the YoMed group were: “Please wear headphones and sit comfortably for the next 15 min of the meditation intervention, with full involvement and no physical shaking. Signal me to start when you’re ready.” The “preparation instructions” before the intervention for the Control group were: “Please wear headphones and sit comfortably for the next 15 min of the eye-closing rest, with full involvement and no physical shaking. Signal me to start when you’re ready.” Then follows, we give the YoMed group “YoMed guidelines,” while no guidelines in the Control group. According to the “YoMed guidelines,” the sitting posture for participants is to sit in a chair and gently pushing their knees with both hands to ensure the uprightness of the spine. During the meditation, the “YoMed guidelines” guide the meditators to focus on breathing and relax their body, head and neck through breathing; immerse their consciousness in the sense of stability that breathing brings to the body; completely focus on the self, without any influence from the surrounding environment, and again concentrate on the breathing.

In the pre-test, participants completed a brief demographic questionnaire after arriving at the laboratory. To assess participants’ recent physical activity, they completed the PARS-3. Following this, participants were seated in front of a computer screen and completed the Flanker tasks, during which the hemodynamic changes of the PFC were monitored by fNIRS. At this point the pre-test was completed. Then, the YoMed group completed the first YoMed exercise under the experimenter’s guidance, while the Control group completed the first eye-closing rest. For the convenience of the participants, we adopted the combination of physical and online intervention. The intervention content of the online course and the physical course was the same. The participants arrived at the laboratory on the 5th day to complete the last YoMed exercise, and the Control group completed the last eye-closing rest. After that, data on Flanker tasks, fNIRS, and physical activity were collected.

In addition, each participants’ immersion state was assessed using a manipulation check at the end of each YoMed intervention or eye-closing rest. The manipulation check measure assessed immersion state on a scale of 20. Higher scores represent a better immersion. The first two questions are related to “Attention,” which are, respectively: (1) It is easy for me to concentrate on what I am doing; (2) I’m easily distracted. Questions 3–4 dealt with “Awareness” (Feldman et al., 2007), which are, respectively: (3) I can usually describe how I currently feel in detail; (4) I can accept the thoughts and feelings I have. The fifth question quantifies individuals’ behavior controlling their attention when they are aware of a distraction, referred to as “attention return” (Walach et al., 2006). And the question is: When I notice an absence of mind, I gently return to the experience of the here and now.

The two occasions were separated by 3 days. During the 3 days, the participants completed YoMed exercises or eye-closing rest under the experimenters’ supervision through the “Tencent Meeting” App every day, and the intervention time was controlled within the same time range.

### Data Preprocessing

Functional near-infrared spectroscopy signal preprocessing was performed in NirSpark V1.5.20 (Danyang Huichuang Medical Equipment Co., Ltd.). Original optical density signals were digital bandpass filtered between 0.01 and 0.2 Hz. Relative-change curves for oxy-Hb, deoxy-Hb, and total hemoglobin (total-Hb) concentrations were calculated using the modified Beer-Lambert law. The optical path difference coefficient of each wavelength was six. Finally, relative concentration signal data of oxy-Hb, deoxy-Hb, and total-Hb were obtained. Compared with deoxy-Hb and total-Hb, oxy-Hb better reflects changes in neural activity, and thus is commonly used to determine brain activation during cognitive tasks (Hoshi et al., 2001). Signals from channels 1–9 were averaged to yield the left prefrontal cortex (LPFC) activity, whereas those from channels 11–19 were averaged to yield the right prefrontal cortex (RPFC) activity. The baseline value of relative change was defined as the average of 10 s between each block in the Flanker task.

### Statistical Analyses

Data were exported to Microsoft Excel for Windows and analyzed in IBM SPSS 24.0. An independent sample *t*-test tested the differences in the participants’ characteristics. For the participants’ physical activity level, immersion state, the performances on the behavioral results (i.e., RTs and accuracy), and the fNIRS data, were assessed by repeated-measures analysis of variance, with a group (YoMed group vs. Control group) served as the between-participants variable whereas the time of measurement (Pre-test vs. Post-test) served as the within-participants variable. Simple effect analyses were conducted to assess the potential interaction effects between time and group. If the sphericity assumption was not fulfilled, the Multivariate Test results will be used. The level of statistical significance for all comparisons was set as *p* < 0.05.

## Results

### Demographic Data

A total of 50 participants were included in this intervention. Due to the low accuracy (25%) in the Flanker task, the data of one participant in the Control group was eliminated. Before the intervention, the demographic variables and physical activity levels of the two groups were analyzed (Table 1). There were no significant differences (*p*s > 0.05) between the groups in terms of age, gender, census register, only child or not, height, weight, or body mass index (BMI). These findings indicated that the demographic characteristics of the two groups were sufficiently homogeneous. Besides, our results showed that there was no significant difference in the level of physical activity between the two groups before and after the experiment (*p*s > 0.05).

**TABLE 1:** Subject characteristics.

	YoMed group (*n* = 25)	Control group (*n* = 24)	χ^2^/*t*	*p*
Age (years)	23.80 ± 1.32	23.88 ± 1.33	–0.198	0.844
Gender (male/female)	11/14	11/13	0.017	0.897
Census register (rural/urban)	16/9	13/11	0.490	0.484
Only child or not (yes/no)	10/15	10/14	0.014	0.906
Height (cm)	169.08 ± 8.17	171.08 ± 8.82	–0.825	0.413
Weight (kg)	67.48 ± 23.55	64.38 ± 12.40	0.574	0.569
BMI (kg/m^2^)	23.31 ± 6.47	21.94 ± 3.82	0.901	0.372
Physical activity in Pre-test	24.00 ± 23.07	24.04 ± 19.69	–0.007	0.995
Physical activity in Post-test	20.12 ± 18.68	19.13 ± 15.64	0.202	0.841

*Values are mean ± SD and numbers (proportion) for gender, census register, and only child or not.*

### Results of Manipulation Effectiveness Under Experimental Conditions

**FIGURE 4:**
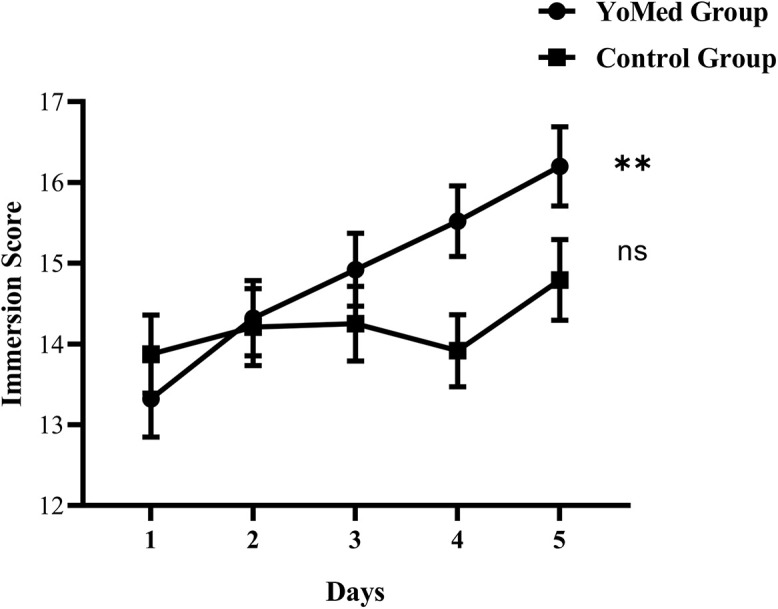
The changing trend of immersion scores in the two groups. The immersion scores of the YoMed group and Control group increased with the extension of the intervention days. The immersion scores evidenced a statistically significant increase in the YoMed group. ^∗∗^*p* < 0.001, with very significant difference, ns *p* > 0.05, no significant difference, and the data is expressed as mean ± standard error.

The immersion scores (Figure 4) of the two groups of participants were analyzed using a repeated-measures ANOVA with a 2 (group: YoMed group vs. Control group)×5 (intervention days) design. The results showed that the main effect of the intervention days was significant, *F*(4,44) = 8.868, *p* < 0.001, and η^2^_p_ = 0.446. The immersion scores of participants increased with the extension of the intervention days. There was no significant difference in the main effect between groups (*p* > 0.05). However, there was a significant interaction effect between group and intervention days. The results of simple effect analysis showed that the scores of YoMed immersion increased significantly with the increase of intervention days, *F*(4,44) = 9.995, *p* < 0.001, and η^2^_p_ = 0.476; while there was no significant change in the immersion scores of participants in the Control group, *F*(4,44) = 1.737, *p* = 0.159, and η^2^_p_ = 0.136. The results of simple effect analysis between the two groups showed that only the 4th day (*p* < 0.05) of intervention and the 5th day (*p* < 0.05) of intervention had significant differences. The results showed that the YoMed practice intervention significantly improved the meditative status of the YoMed group, indicating that the intervention in this study was effective.

The results of three subscales of attention, awareness, and attention return showed that the main effect of intervention days was significant (*ps* < 0.05). It indicates that with the increase of intervention time, the scores of the participants in these three subscales of the immersion state have significantly increased. There was a significant interaction between intervention days and group in terms of awareness and attention return (*ps* < 0.05), indicating that with the intervention advances, only the two aspects of awareness, and attention return showed inter-group differences.

### Flanker Task Accuracy

The Flanker task accuracy was analyzed using a repeated-measures ANOVA (Table 2) with a two (group: YoMed group vs. Control group)×2 (time: Pre-test vs. Post-test)×2 (task type: congruent vs. incongruent) design. The main effect of the task type was statistically significant, *F*(1,47) = 37.521, *p* < 0.001, and η^2^_p_ = 0.444. The accuracy of participants on the congruent task (99.106 ± 1.159%) was significantly higher than that on the incongruent task (93.605 ± 7.413%). The main effect of group was significant, *F*(1,47) = 5.968, *p* = 0.018, and η^2^_p_ = 0.113. The accuracy of participants in the YoMed group (97.555 ± 3.033%) was significantly higher than that of the Control group (95.106 ± 7.772%). The interaction between time and group was significant, *F*(1,47) = 7.167, *p* = 0.010, and η^2^_p_ = 0.132. The results of simple effect analysis showed that the accuracy of participants in the YoMed group (97.940 ± 2.644%) was significantly higher than that of the Control group (94.417 ± 8.610%) in the Post-test, *p* = 0.003. However, there was no significant difference in the accuracy between the two groups in the Pre-test, *p* = 0.199.

**TABLE 2:** The accuracy (%) and RTs (ms) on the Flanker test (M ± SD).

Index	Task type	YoMed group (*n* = 25)		Control group (*n* = 24)	
			
		Pre-test	Post-test	Pre-test	Post-test
Accuracy (%)	Congruent task	99.176 ± 0.869	99.672 ± 0.537	98.512 ± 1.680	99.038 ± 1.013
	Incongruent task	95.164 ± 3.733	96.208 ± 2.780	93.079 ± 8.819	89.796 ± 10.291
RT (ms)	Congruent task	474 ± 45	464 ± 38	462 ± 56	433 ± 52
	Incongruent task	578 ± 58	548 ± 41	561 ± 68	512 ± 60

The interaction of time, task type and group is significant, *F*(1,47) = 9.318, *p* = 0.004, and η^2^_p_ = 0.165. The simple effect analysis showed that in the congruent task, the accuracy of participants in the YoMed group (99.672 ± 0.537%) was significantly higher than that of the Control group (99.038 ± 1.013%) in the Post-test, *p* = 0.008; however, there was no significant difference in accuracy between the two groups (*p* = 0.087) in the Pre-test. After the intervention in inconsistent tasks, the accuracy in the YoMed group (96.208 ± 2.780%) was significantly higher than that in the Control group (89.796 ± 10.291%), *p* = 0.004; In the Pre-test, there was no significant difference between the two groups (*p* = 0.283).

### Flanker Task RTs

The Flanker task RTs were analyzed using a repeated-measures ANOVA (Table 2) with a 2 (group: YoMed group vs. Control group)×2 (time: Pre-test vs. Post-test)×2 (task type: congruent vs. incongruent) design. Our results shows that the main effect of time was statistically significant, *F*(1,47) = 23.734, *p* < 0.001, and η^2^_p_ = 0.336. The RTs in the Post-test (489 ± 65 ms) was significantly faster than that in the Pre-test (519 ± 76 ms). The main effect of task type was statistically significant, *F*(1,47) = 741.172, *p* < 0.001, and η^2^_p_ = 0.940. The RTs of participants on the congruent task (459 ± 50 ms) were significantly faster than those on the incongruent task (550 ± 62 ms). The interaction between time and the task type was statistically significant, *F*(1,47) = 18.250, *p* < 0.001, and η^2^_p_ = 0.280. The main effect of the group and the other interactions were not statistically significant (*ps* > 0.05).

### Functional Near-Infrared Spectroscopy (fNIRS) Results

**FIGURE 5:**
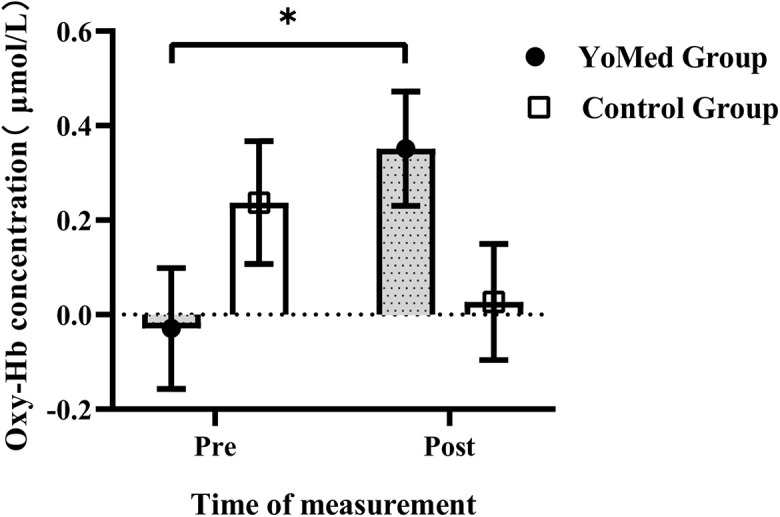
The cerebral oxy-Hb concentration in the prefrontal cortex (PFC) between YoMed and Control groups at pre- and post-test. The cerebral oxy-Hb concentration in the prefrontal cortex for the YoMed group at post-test was significantly higher than those at pre-test. While there was no statistically significant change in the Control group. ^∗^*p* < 0.05, with significant difference. Data are expressed as mean ± standard error.

The oxy-Hb data were analyzed using a repeated-measures ANOVA with a 2 (group: YoMed group vs. Control group)×2 (time: Pre-test vs. Post-test)×2 (task type: congruent task vs. incongruent task)×2 (brain region: left brain vs. right brain) design. The result shows that the interaction between time and group was significant, *F*(1,47) = 5.435, *p* = 0.024, and η^2^_p_ = 0.104. As is shown in Figure 5. The results of the simple effect test showed that the cerebral oxy-Hb variation of the YoMed group was significantly higher after the intervention (0.351 ± 0.674 μmol/L) than before the intervention (0.029 ± 0.672 μmol/L), *p* = 0.037. However, there was no significant difference in cerebral oxy-Hb variation in the Control group before and after intervention (*p* = 0.252). Other main effects and interactions were not significant (*p* > 0.05).

## Discussion

Our research shows that YoMed intervention can improve young adults’ performance in a Flanker task and enhance brain activation in the PFC during the cognitive task. This indicates that YoMed can improve the inhibitory control of young adults and enhance the activation of the brain regions related to cognitive functions.

The results of the manipulation check showed that participants were capable of entering the YoMed state smoothly after the 5-day practice. They need to constantly be aware of the quality of their attention in order to maintain the attentive state during YoMed practice (Lutz et al., 2008). Participants’ attention may deviate during YoMed, which requires participants to detect this deviation and return to their previous state of concentration. Our results revealed that these two skills of regulating attention have been increased in the process of YoMed intervention. However, no significant interaction between intervention days and group was found in “attention.” This may be related to the relatively short duration of YoMed practice. A previous study has shown that high self-reported scores of attention absorption by meditators are positively correlated with the accumulated training time, which can be regarded as the accumulation of attention or attention control (Grant et al., 2013). And the “attention” scores of participants in the YoMed group in our study showed an upward trend over time. Therefore, this change may be significant only if prior experience was accumulated.

The results from the Flanker task showed that there was no significant difference in the accuracy between the two groups before the intervention test. However, after the intervention, the accuracy for the YoMed group was higher than those of the Control group during the congruent task and incongruent task, and there were statistically significant differences. This finding suggests that the YoMed improved Flanker task performance in young adults. The results are consistent with previous studies. An 8-week randomized controlled trial conducted by Sharma et al. (2014) showed that Sahaja yoga improved attention span, increased concentration, and faster visual-motor speed in healthy subjects. Saoji et al. (2017) conducted a randomized, bidirectional cross-over study to investigate the effects of a YoMed technique called Psychic Sound Resonance (MSRT) on the cognitive functioning that requires sustained attention and activation and inhibition of the speed of information processing of university medical students. It was found that YoMed techniques may have a positive effect on college medical students’ performance on cognitive tasks and reduce the effects of anxiety and stress on them. Consistent with previous studies, participants in the YoMed group in this study also showed significantly higher accuracy than those in the Control group in the consistent task. On the basis of previous studies, we further found that YoMed can improve the higher executive function (including inhibitory control) which is a kind of higher cognitive function. In other words, YoMed practice also has a positive effect on participants’ ability to perform inhibitory controls.

The results from the Flanker task showed that there was no significant difference in the RTs between the two groups before and after the intervention, but the RTs was improved in both groups, which indicated that YoMed practice did not influence the cognitive processing speed of young adults more directly. This improvement in RT may be due to practice effects in doing the task. This is inconsistent with some of the previous studies (Tang et al., 2007; Fan et al., 2014). It is worth noting that both studies used a holistic mind-body conditioning training system, which differs from traditional YoMed. Previous research has shown that different meditation techniques often involve different practices with great heterogeneity. This may have different effects on the brain (Hernandez et al., 2016) and in turn affect individual behavior. Based on the above views, we found that the YoMed practice adopted in this study requires participants to focus on their breathing and focus their attention to follow the guidance words to achieve a state of highly focused immersion, and some dynamic changes in the external environment will not have an impact on participants. This suggests that participants in YoMed without having to react to unpredictable changes in the external environment, but simply focus on the stable environment in meditation, which may not have more impact on the reaction speed of participants. Therefore, we speculate that the reason why this study differs from the above studies in response time index may be that different YoMed practice methods were adopted in this study (Hernandez et al., 2020).

Yoga meditation techniques involve repeatedly focusing attention on objects while alternately acknowledging and releasing distracting thoughts and emotions. It may include proprioceptive sensations caused by body posture or breathing (Froeliger et al., 2012b). Integration of cognitive and motor control is mediated by anatomical connections between units of the cerebellum and areas of the PFC (Oakley and Evans, 2014). The practice of YoMed can stimulate frontal neuroplasticity through the cognitive and motor skill learning involved in such practice. Yoga meditators can unify the body and mind and improve cognition by developing higher mindfulness and self-discipline. YoMed is simple and can enhance brain plasticity so that the cognitive function of young adults trained in YoMed can be improved (Froeliger et al., 2012a).

The fNIRS results demonstrated that, compared with the Control group, the participants who received the YoMed intervention had higher oxy-Hb concentrations in the PFC when they completed the Flanker task. In this study, an increase in oxy-Hb concentration was found to reflect greater cortical activation, which showed that YoMed significantly enhanced the activation of the PFC during the Flanker task. This finding is consistent with previous studies. For example, Froeliger et al. (2012a) study found that participants in the YoMed group showed greater volume in the frontal, limbic, temporal, occipital, and cerebellar regions, with fewer reports of cognitive failure. Their results suggest that the YoMed can lead to local structural changes in the brain that are reflected in better cognitive ability (Froeliger et al., 2012a). Similarly, our study found an increase in blood oxygen levels in the PFC, suggesting that YoMed elicits temporary changes in cognitive functioning in the brain.

The PFC as a whole plays a key role in organizing behavior, language, and cognitive behavior. Cognitive function in the adult PFC can lead to the highest temporal integration of expression of language and intellectual performance and is considered to be the culmination of biological processes (Fuster, 2002). The study found that YoMed increased frontal lobe activation during control tasks. A study led by Fox et al. (2014) also found that long-term meditators had greater frontal volume. YoMed has been found to cause expansion of the inferior frontal area; the reason may be that it allows practitioners to suppress unwanted thoughts and control their attention (Hernandez et al., 2020). In addition, meditation also showed better emotional dissociation and self-control, which is mediated by the orbitofrontal and ventromedial frontal regions.

The study is not without limitations. Firstly, our research found that YoMed practice had a temporary state impact on young adults’ inhibitory control function. Future research should continue to explore the functional changes of inhibition control of young adults caused by YoMed practice from the perspective of long-term intervention. Secondly, the spectral probe used in this study covered only the PFC. And important subcortical areas are very difficult to detect. Third, the YoMed adopted in this study belongs to focused attention meditation, which uses the seated posture in the chair to meditate according to the “preparation instructions” and guidelines. However, this is different from some YoMed (use other postures or different guidelines), or OM meditation. Different forms of meditation may lead to activation in different areas of the brain. In the future, we should focus on using different meditation methods to intervene and explore whether different meditation methods will have different effects on the brain. Finally, the intervention needs to be tested in other populations.

The value of this study is several fold. First, this study further reveals the important role of YoMed in promoting inhibition and control, and enriches the relevant literature. Second, the results of this study contribute to understanding the effects of YoMed on behavioral performance and brain activity. It is easy to learn and can be easily integrated into the busy schedule of participants and provides evidence that YoMed may promote inhibitory control function in young adults. In addition, the results of this study have implications for designing YoMed programs for young adults to improve their inhibitory control function and enhance their cognitive function.

## Conclusion

Yoga meditation has a temporary and positive effect on the cognitive function of young adults. This was shown by improved performance on the Flanker task, and the activation of PFC also showed an immediacy increase in participants who received the YoMed intervention. YoMed is easy to use and a safe exercise that is practical for young adults and is worth popularizing among young adults.

## Data Availability Statement

The original contributions presented in the study are included in the article/supplementary material, further inquiries can be directed to the corresponding author.

## Ethics Statement

The studies involving human participants were reviewed and approved by School of Public Health, Shandong University. The patients/participants provided their written informed consent to participate in this study.

## Author Contributions

DJ, ZL, and GS: conceptualization and formal analysis. DJ, ZL, and GS: writing–review and editing. DJ: software, data curation, and fNIRS data analysis. GS: resources, project administration, and funding acquisition. DJ and ZL: writing–original draft preparation. All authors contributed to the article and approved the submitted version.

## Conflict of Interest

The authors declare that the research was conducted in the absence of any commercial or financial relationships that could be construed as a potential conflict of interest.

## Publisher’s Note

All claims expressed in this article are solely those of the authors and do not necessarily represent those of their affiliated organizations, or those of the publisher, the editors and the reviewers. Any product that may be evaluated in this article, or claim that may be made by its manufacturer, is not guaranteed or endorsed by the publisher.
